# The role of three glucose/lipid composite indices (CHG, TYG, and AIP) in predicting carotid plaque and fatty liver outcomes: a retrospective cohort study

**DOI:** 10.3389/fendo.2025.1686931

**Published:** 2025-10-14

**Authors:** Dan Li, Zhaohui Xu, Feifei Wang, Yinqin Hu, Xinyu Zhang, Jiahui Yang, Qiqi Wan, Ning Zhang, Yongming Liu

**Affiliations:** ^1^ Shuguang Hospital Affiliated to Shanghai University of Traditional Chinese Medicine, Shanghai, China; ^2^ Ansteel Group General Hospital, Anshan Liaoning, China; ^3^ Anhui Hospital of Shuguang Hospital Affiliated to Shanghai University of Traditional Chinese Medicine, Hefei, Anhui, China

**Keywords:** carotid plaque, fatty liver, TyG, CHG, AIP

## Abstract

**Background:**

Carotid plaque and fatty liver disease, as important target organ damages of metabolic disorders, have undergone a steady increase in prevalence. Cholesterol, high-density lipoprotein, and glucose index (CHG), triglyceride–glucose index (TYG), and atherogenic index of plasma (AIP) are tools for assessing metabolic abnormalities. This research aimed to evaluate the potential of three indicators in predicting carotid plaque and fatty liver.

**Methods:**

This study is based on longitudinal health examination data from workers at Ansteel Group in China in 2019. The follow-up period was five years, with the outcomes being the occurrence of carotid plaque or fatty liver events. Multivariate Cox regression analysis was used to examine the relationship between CHG, TYG, and AIP with the outcomes of carotid artery plaque and fatty liver. We used restricted cubic spline (RCS) curves to analyze the dose-response relationship between the three indices and the outcomes. We employed receiver operating characteristic (ROC) curves to evaluate the predictive ability of these indices. Finally, we also conducted subgroup analyses.

**Results:**

Carotid plaque events developed in 659 workers (18.40%), and fatty liver in 375 workers (10.47%) during the follow-up period. Cox analysis revealed that the three indices were correlated with carotid plaque (Q3 vs Q1, CHG: HR 2.13, *P* < 0.001; TYG: HR 1.20, *P* = 0.006; AIP: HR 1.95, *P* < 0.001) and fatty liver (Q3 vs Q1, CHG: HR 2.46, *P* < 0.001; TYG: HR 1.75, *P* < 0.001; AIP: HR 3.47, *P* < 0.001). RCS indicated that the three indices were linearly related to carotid plaque and nonlinearly (inverted L-shaped) related to fatty liver. ROC curve analysis revealed that CHG had a stronger predictive ability for carotid plaque outcomes, while TYG had a stronger predictive ability for fatty liver. Subgroup analysis results showed that gender and BMI interacted with the three indicators in relation to outcomes.

**Conclusions:**

Our research found that CHG, TYG, and AIP were positively correlated with carotid plaque and fatty liver. Moreover, CHG demonstrated superior predictive ability for carotid plaque outcomes, whereas TYG demonstrated better performance for fatty liver outcomes.

## Introduction

With improvements in living standards, metabolic disorders have increasingly become a global public health concern. Carotid plaque and fatty liver disease, which are important target organ damages, continue to rise in prevalence. Carotid plaque is a major risk factor for cardiovascular disease (CVD), with a prevalence rate as high as 40% in people aged 40 and above (1). Fatty liver disease is closely related to various metabolic disorders, with a prevalence rate of 20% to 30% in the general population (2). Research has confirmed that liver fat content is associated with increased carotid intima-media thickness and shares core pathophysiological mechanisms, including chronic low-grade inflammation, insulin resistance (IR), lipid metabolism disorders, and endothelial dysfunction (3–6). The “liver-vascular axis” theory proposes that fatty liver disease is not only a local liver lesion, but also an independent risk factor for systemic vascular disease (7, 8). Within this framework, carotid plaque (a window marker of systemic atherosclerosis) interacts with fatty liver disease to jointly exacerbate metabolic disorders and vascular damage, forming a vicious cycle of “metabolism-liver-vessels” (9–11).

Clinical studies indicate that impaired glucose metabolism promotes the accumulation of atherogenic lipoproteins, synergistically accelerating vascular damage (12). Traditional single lipid or glucose markers struggle to capture the complex interactions between glucose and lipid metabolism, and their predictive power for complex outcomes driven by multiple metabolic factors is often limited. Integrating lipid and glucose data, however, better defines metabolic characteristics and vascular risk profiles (13). Consequently, there is an urgent clinical need for composite indicators that integrate multidimensional information and more sensitively reflect the overall state of metabolic dysfunction. Triglyceride-glucose index (TYG) and plasma atherosclerosis index (AIP) have been proven to have good predictive value for cardiovascular and metabolic disease risk (11, 14, 14). However, the cholesterol, high-density lipoprotein, and glucose index (CHG) is an emerging glucose-lipid composite index that is rarely studied in metabolic diseases (15, 16). There are currently no comparative studies on the predictive potential of these three indicators for carotid plaque and fatty liver. This research aimed to investigate the association between CHG/TYG/AIP and the above-mentioned target organ damage through a cohort study, and to evaluate its predictive ability.

## Methods

### Study population and design

Derived from longitudinal health examinations of steelworkers at Ansteel Group (Anshan, China), this retrospective cohort study analyzed data from a series of health assessments. All employees of the company undergo annual health examinations as mandated by Chinese labor regulations. The study collected electronic health check-up data (including physical examinations, hematological tests, ultrasound scans, etc.) from Ansteel Group General Hospital for employees between 2019 and 2023, and constructed a longitudinal cohort database. The study was approved by the Ethics Committee of Ansteel Group General Hospital, approval number: 2025-0045. Patient information was de-identified, and the study complies with the principles of the Declaration of Helsinki.

**Figure 1:**
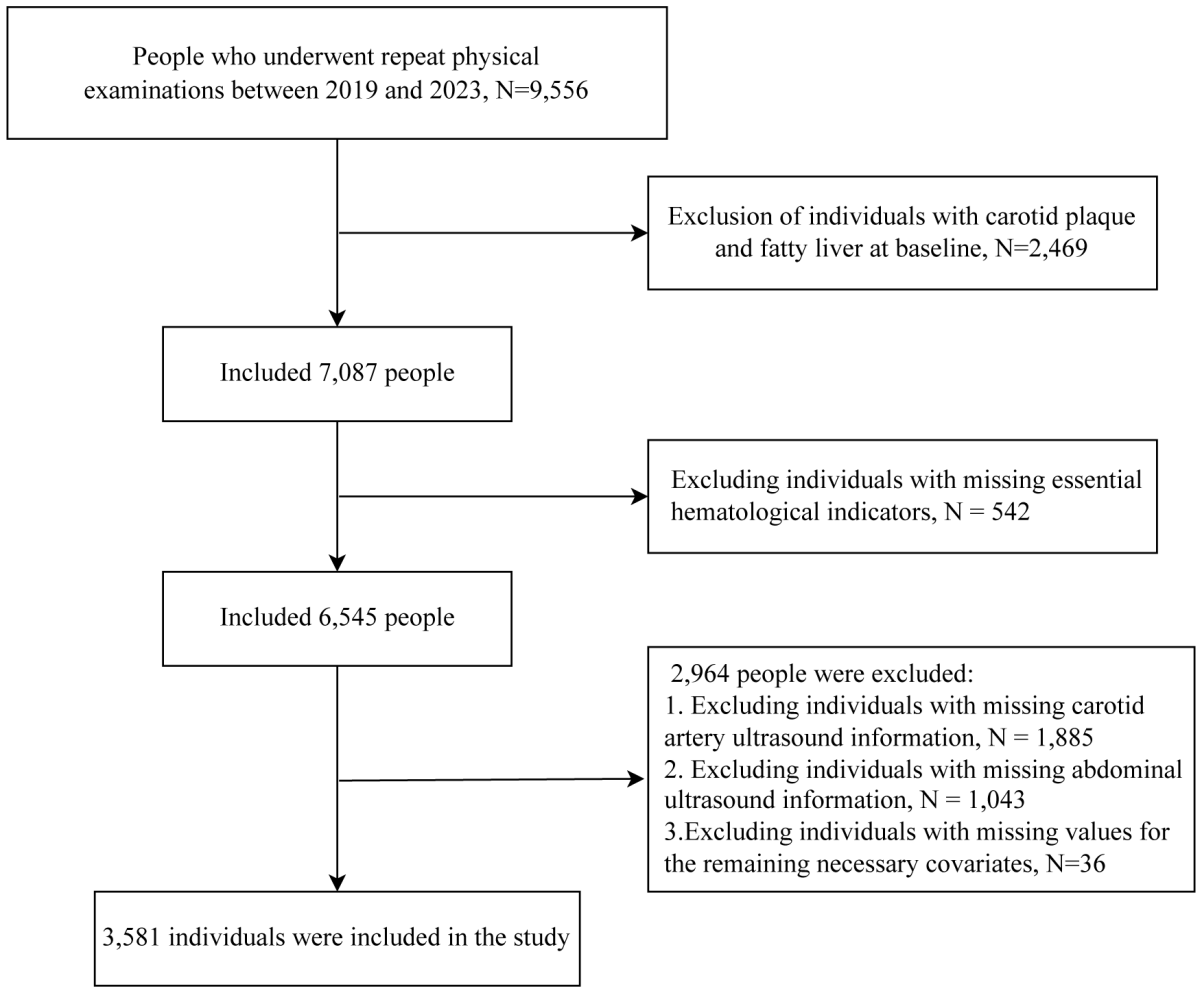
Flow chart.

The study selected 9,556 individuals with serial physical examination data between 2019 and 2023. We excluded 2,469 individuals who had carotid plaque and fatty liver at baseline. After excluding 542 individuals with incomplete blood glucose or lipid data, 2,928 individuals lacking complete annual carotid and abdominal ultrasound records during the study period, and 36 individuals with incomplete covariate data, a total of 3,581 individuals were ultimately included in this study. The inclusion criteria are shown in **Figure 1**.

### Definitions of CHG, TYG, and AIP indices

All indicators were obtained from peripheral blood samples taken ≥ 8 hours after fasting in the morning. Serum triglyceride (TG), total cholesterol (TC), and fasting blood glucose (FBG) levels were measured using enzyme-linked immunosorbent assays, while high-density lipoprotein cholesterol (HDL-C) was determined via chemical precipitation (17). The indices were calculated as follows: CHG index = Ln [TC (mg/dL) × FBG (mg/dL)/2 × HDL (mg/dL)] (16); TyG index = ln (TG (mg/dL) × FBG (mg/dL)/2) (18); AIP = Log [TG (mmol/L)/HDL-C (mmol/L)] (19). Participants were stratified by tertiles: CHG: Q1 (<4.94), Q2 (4.94–5.21), Q3 (>5.21); TyG: Q1 (<8.39), Q2 (8.39–8.99), Q3 (>8.99); AIP: Q1 (<0.15), Q2 (0.15–0.45), Q3 (>0.45).

### Endpoint assessment

The primary endpoint of this study was the occurrence of carotid plaque or fatty liver. Carotid plaque was detected by carotid ultrasonography with a Philips i U22 model color Doppler ultrasound diagnostic system and accompanying software, a line array probe was taken, and the frequency was set from 7 to 11.2 MHz. Plaque diagnosis required: (a) focal wall thickening ≥0.5 mm or exceeding 50% of surrounding CIMT; (b) lumen-protruding foci; or (c) CIMT >1.5 mm in any carotid arterial segment (20). Fatty liver was defined as the presence of hepatic steatosis as shown by abdominal ultrasound. A 3.5 MHz convex array probe is used for abdominal color Doppler ultrasound detection. Color Doppler ultrasound was performed by a team of two experienced physicians. Fatty liver degeneration was diagnosed by abdominal ultrasound when ≥2 of these features were present: hepatic parenchymal brightness, deep attenuation, bright vessel walls, hepatorenal echogenicity contrast, or gallbladder wall blurring (21, 22).

### Included variables

Demographic characteristics included gender, age, and body mass index (BMI). Laboratory test data include heart rate, systolic blood pressure, diastolic blood pressure, albumin, FBG, TG, HDL-C, TC, low-density lipoprotein cholesterol (LDL-C), creatinine, platelet (PLT), hemoglobin (Hb), white blood cells (WBC), neutrophils, lymphocytes, monocytes, aspartate transaminase (AST) and gamma-glutamyl transpeptidase (GGT), and alanine transaminase (ALT).

### Statistical analysis

We used descriptive statistical methods to summarize the baseline characteristics of the study participants. The normality of continuous variables was assessed using the Shapiro-Wilk test and visual inspection of Q-Q plots. Continuous variables were normally distributed or approximately normally distributed, expressed as mean ± standard deviation. Categorical variables were expressed as N (%). Intergroup differences were analyzed using analysis of variance (ANOVA) for continuous variables and χ² tests for categorical variables. Multivariate Cox proportional hazards regression was performed to assess associations between CHG, TYG, and AIP levels and carotid plaque/fatty liver. Results are presented as hazard ratios (HR) with corresponding 95% confidence intervals (CI). Two models were adjusted. Model 1 adjusted for sex, age, and BMI. Model 2 additionally adjusted for diastolic blood pressure, systolic blood pressure, heart rate, creatinine, albumin, white blood cells, platelets, hemoglobin, neutrophils, lymphocytes, monocytes, gamma-glutamyl transpeptidase, alanine transaminase, and aspartate transaminase. All variables passed multicollinearity tests, with variance inflation factor values below 5 (**Supplementary Table S1**). Using the Cox regression model, we established restricted cubic splines (RCS) and assessed dose-response relationships using the likelihood ratio test.

Kaplan-Meier curves were generated to visualize cumulative outcome risks over an observation period of 1 to 5 years. Receiver operating characteristic (ROC) analysis was conducted to compare area under the curve (AUC) values of the three indices. Additionally, the AUC values underwent DeLong’s test. Finally, subgroup analyses were performed by age, gender, and BMI to evaluate predictive ability across populations.

This research used R (4.3.0). The study considered two-sided p-value < 0.05 to be statistically significant.

## Results

### Baseline characteristics

**Table 1** presents baseline characteristics grouped by carotid plaque and fatty liver status. The cohort had a mean age of 45.82 ± 9.46 years with 47.28% males. Over the 5-year follow-up period, 659 (18.40%) workers developed carotid plaque, and 375 (10.47%) workers developed fatty liver. Compared to those without carotid plaque, affected individuals exhibited higher mean age, greater male predominance, elevated BMI, increased blood pressure, higher CHG/TYG/AIP indices, elevated blood glucose, and poorer lipid profiles. Similar trends were observed in the fatty liver group. Both outcome groups also demonstrated elevated inflammatory markers and liver enzymes.

**Table 1:** Baseline characteristics of study population.

Variables	Total (n = 3581)	Carotid plaque		*P*-value	Fatty liver		*P*-value
		No (n = 2922)	Yes (n = 659)		No (n = 3206)	Yes (n = 375)	
CHG, Mean ± SD	5.10 ± 0.33	5.06 ± 0.31	5.27 ± 0.34	< 0.001	5.08 ± 0.33	5.22 ± 0.30	< 0.001
TYG, Mean ± SD	8.74 ± 0.72	8.69 ± 0.71	9.00 ± 0.70	< 0.001	8.70 ± 0.71	9.07 ± 0.68	< 0.001
AIP, Mean ± SD	0.31 ± 0.33	0.29 ± 0.32	0.43 ± 0.31	< 0.001	0.30 ± 0.33	0.45 ± 0.29	< 0.001
Male, N (%)	1693 (47.28)	1217 (41.65)	476 (72.23)	< 0.001	1525 (47.57)	168 (44.80)	0.310
Age, Mean ± SD	45.82 ± 9.46	44.03 ± 8.66	53.73 ± 8.76	< 0.001	45.74 ± 9.45	46.45 ± 9.49	0.173
BMI, N (%)				< 0.001			< 0.001
<25 kg/m^2^	1756 (58.71)	1492 (60.95)	264 (48.62)		1630 (60.98)	126 (39.62)	
≥25 kg/m^2^	1235 (41.29)	956 (39.05)	279 (51.38)		1043 (39.02)	192 (60.38)	
Laboratory indicators, Mean ± SD							
SBP, mmHg	126.00 ± 13.60	124.00 ± 12,74	138.00 ± 15.49	< 0.048	126.00 ± 14.65	133.00 ± 17.59	< 0.001
DBP, mmHg	68.88 ± 26.55	68.69 ± 25.90	69.71 ± 29.28	0.415	68.66 ± 26.37	70.87 ± 28.11	0.168
Heart Rate, BPM	69.32 ± 25.43	69.67 ± 25.47	67.73 ± 25.20	0.114	69.58 ± 25.21	67.09 ± 27.14	0.107
Albumin, g/L	45.18 ± 5.13	45.30 ± 4.87	44.67 ± 6.11	0.005	45.32 ± 5.22	43.98 ± 4.07	< 0.001
FBG, mmol/L	5.72 ± 1.39	5.61 ± 1.25	6.20 ± 1.82	< 0.001	5.68 ± 1.36	6.06 ± 1.60	< 0.001
TC, mmol/L	5.16 ± 0.94	5.14 ± 0.92	5.23 ± 1.03	0.036	5.14 ± 0.93	5.29 ± 0.99	0.004
TG, mmol/L	1.77 ± 1.73	1.70 ± 1.66	2.11 ± 2.02	< 0.001	1.72 ± 1.68	2.28 ± 2.09	< 0.001
HDL-C, mmol/L	1.60 ± 0.35	1.63 ± 0.34	1.47 ± 0.36	< 0.001	1.61 ± 0.35	1.53 ± 0.30	< 0.001
LDL-C, mmol/L	3.10 ± 0.80	3.08 ± 0.79	3.20 ± 0.82	< 0.001	3.09 ± 0.79	3.22 ± 0.80	0.003
Creatinine, μmol/l	64.16 ± 34.42	62.43 ± 25.04	71.87 ± 60.01	< 0.001	63.96 ± 29.95	65.89 ± 60.39	0.303
WBC, ×10^9^/L	5.91 ± 1.58	5.88 ± 1.55	6.02 ± 1.69	0.047	5.87 ± 1.59	6.23 ± 1.49	< 0.001
Hb, g/L	138.18 ± 15.65	136.65 ± 15.67	145.00 ± 13.62	< 0.001	137.96 ± 15.80	140.03 ± 14.19	0.015
PLT, ×10^9^/L	231.96 ± 54.43	235.30 ± 53.34	216.99 ± 56.72	< 0.001	231.75 ± 54.91	233.76 ± 50.12	0.500
Neutrophils, ×10^9^/L	3.42 ± 1.18	3.41 ± 1.17	3.44 ± 1.21	0.573	3.40 ± 1.18	3.61 ± 1.12	< 0.001
Lymphocytes, ×10^9^/L	2.00 ± 0.58	1.99 ± 0.57	2.06 ± 0.65	0.007	1.99 ± 0.58	2.12 ± 0.57	< 0.001
Monocytes, ×10^9^/L	0.33 ± 0.11	0.33 ± 0.11	0.35 ± 0.13	< 0.001	0.33 ± 0.11	0.34 ± 0.11	0.044
AST, U/L	27.73 ± 22.96	27.59 ± 23.43	28.36 ± 20.78	< 0.001	27.29 ± 22.76	31.49 ± 24.34	< 0.001
ALT, U/L	20.28 ± 11.76	19.95 ± 11.11	21.78 ± 14.23	< 0.001	20.11 ± 11.60	21.75 ± 13.00	0.011
GGT, U/L	40.83 ± 13.42	40.70 ± 13.63	41.37 ± 12.40	0.251	40.10 ± 12.67	40.91 ± 13.50	0.270

BMI, Body Mass Index, SBP, Systolic Blood Pressure, DBP, Diastolic Blood Pressure, FBG:Fasting Blood Glucose, WBC, White Blood Cells, Hb, Hemoglobin, PLT, Platelets, AST, Aspartate Aminotransferase, ALT, Alanine Aminotransferase, GGT, Gamma-Glutamyl Transferase.

### Relationship between CHG, TYG, and AIP and outcomes

**Table 2** demonstrates the relationship between the three indices and outcomes related to carotid artery plaques and fatty liver. When analyzed continuously, each 1-unit increase in CHG corresponded to 1.13-fold higher carotid plaque risk (95% CI 1.63–2.79, *P* < 0.001) and 1.46-fold higher fatty liver risk (95% CI 1.68–3.58, *P* < 0.001).

**Table 2:** The relationship between the indices and the outcomes.

Variables	Unadjusted	*P-*value	Model 1	*P-*value	Model 2	*P-*value	AUC
Carotid plaque							
CHG, continuous	4.55 (3.73~5.56)	<0.001	2.26 (1.78~2.85)	<0.001	2.13 (1.63~2.79)	<0.001	0.678
Q1	1(Ref)		1(Ref)		1(Ref)		
Q2	1.96 (1.55~2.49)	<0.001	1.34 (1.05~1.71)	0.019	1.23 (0.94~1.61)	0.124	
Q3	3.76 (3.03~4.68)	<0.001	1.96 (1.55~2.49)	<0.001	1.85 (1.42~2.40)	<0.001	
* P* for trend	1.94 (1.75~2.14)	<0.001	1.42 (1.26~1.58)	<0.001	1.39 (1.23~1.57)	<0.001	
TYG, continuous	1.62 (1.48~1.78)	<0.001	1.28 (1.15~1.43)	<0.001	1.20 (1.05~1.37)	0.006	0.634
Q1	1(Ref)		1(Ref)		1(Ref)		
Q2	2.02 (1.61~2.52)	<0.001	1.35 (1.07~1.70)	0.010	1.36 (1.06~1.74)	0.017	
Q3	2.90 (2.34~3.58)	<0.001	1.74 (1.39~2.19)	<0.001	1.61 (1.25~2.08)	<0.001	
*P* for trend	1.65 (1.50~1.83)	<0.001	1.32 (1.18~1.47)	<0.001	1.26 (1.11~1.42)	<0.001	
AIP, continuous	3.19 (2.56~3.97)	<0.001	2.04 (1.57~2.64)	<0.001	1.95 (1.45~2.63)	<0.001	0.632
Q1	1(Ref)		1(Ref)		1(Ref)		
Q2	1.88 (1.51~2.34)	<0.001	1.43 (1.14~1.79)	0.002	1.46 (1.14~1.87)	0.003	
Q3	2.66 (2.16~3.27)	<0.001	1.77 (1.41~2.22)	<0.001	1.66 (1.29~2.15)	<0.001	
* P* for trend	1.59 (1.45~1.76)	<0.001	1.31 (1.18~1.46)	<0.001	1.27 (1.12~1.43)	<0.001	
Fatty liver							
CHG, continuous	2.88 (2.19~3.78)	<0.001	3.24 (2.33~4.50)	<0.001	2.46 (1.68~3.58)	<0.001	0.645
Q1	1(Ref)		1(Ref)		1(Ref)		
Q2	2.39 (1.76~3.24)	<0.001	2.54 (1.82~3.56)	<0.001	2.02 (1.43~2.86)	<0.001	
Q3	3.24 (2.41~4.34)	<0.001	3.79 (2.69~5.32)	<0.001	2.53 (1.76~3.64)	<0.001	
*P* for trend	1.70 (1.49~1.94)	<0.001	1.84 (1.57~2.14)	<0.001	1.52 (1.28~1.80)	<0.001	
TYG, continuous	1.79 (1.59~2.02)	<0.001	1.82 (1.58~2.09)	<0.001	1.75 (1.49~2.05)	<0.001	0.657
Q1	1(Ref)		1(Ref)		1(Ref)		
Q2	3.05 (2.19~4.25)	<0.001	3.17 (2.23~4.50)	<0.001	3.03 (2.05~4.48)	<0.001	
Q3	4.32 (3.14~5.95)	<0.001	4.58 (3.22~6.54)	<0.001	4.27 (2.85~6.40)	<0.001	
*P* for trend	1.89 (1.65~2.17)	<0.001	1.95 (1.67~2.27)	<0.001	1.87 (1.57~2.22)	<0.001	
AIP, continuous	3.63 (2.73~4.83)	<0.001	4.12 (2.95~5.76)	<0.001	3.47 (2.35~5.14)	<0.001	0.628
Q1	1(Ref)		1(Ref)		1(Ref)		
Q2	2.50 (1.82~3.43)	<0.001	2.59 (1.85~3.63)	<0.001	2.29 (1.57~3.34)	<0.001	
Q3	3.75 (2.77~5.07)	<0.001	4.14 (2.94~5.83)	<0.001	3.60 (2.44~5.31)	<0.001	
*P* for trend	1.83 (1.60~2.09)	<0.001	1.94 (1.66~2.26)	<0.001	1.81 (1.52~2.17)	<0.001	

Model 1 adjusted for age, gender, and BMI.

Model 2 further adjusted for heart rate, diastolic blood pressure, systolic blood pressure, creatinine, albumin, white blood cells, platelets, hemoglobin, neutrophils, lymphocytes, monocytes, gamma-glutamyl transpeptidase, alanine transaminase, and aspartate transaminase.

**Figure 2:**
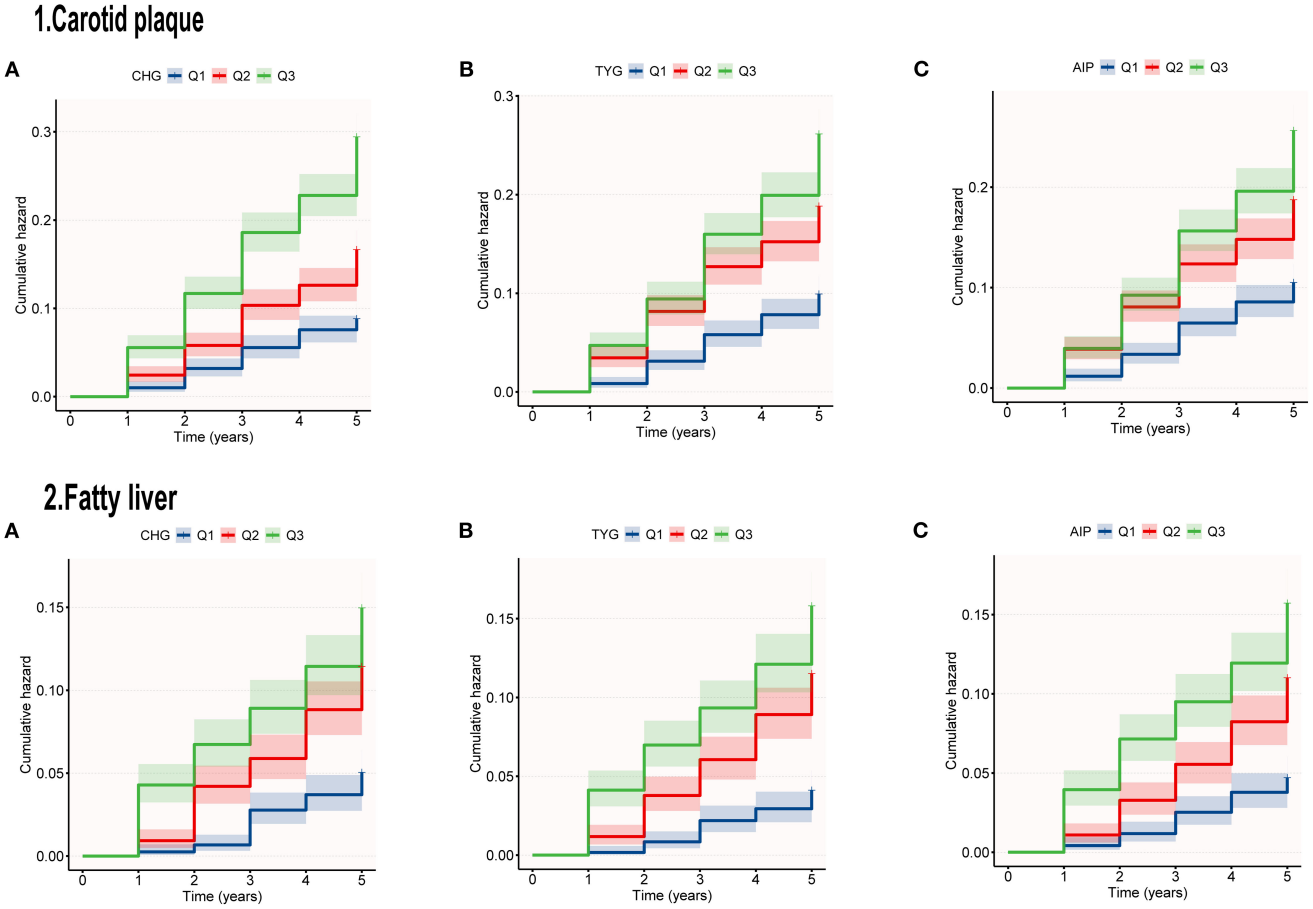
Kaplan–Meier survival curves for cumulative outcomes risk by CHG **(A)**, TYG **(B)**, AIP **(C)**.

Categorical analysis revealed that compared with the Q1 group, the CHG-Q3 group exhibited significantly elevated risks for both outcomes (carotid plaque: adjusted HR = 1.85, P<0.001; fatty liver: HR = 2.53, P<0.001). TYG tertiles showed significant positive correlations with both outcome risks (P < 0.001). For carotid plaque, the adjusted risk ratio in the Q3 group compared to the Q1 group was 1.61. For fatty liver, the adjusted HR in the Q3 group compared to the Q1 group was 4.27. Results from the AIP study were consistent, with adjusted risk ratios for carotid plaque and fatty liver in the Q3 group (compared to Q1) being 1.95 (95% CI: 1.45–2.63, P < 0.001) and 3.47 (95% CI: 2.35–5.14, P < 0.001), respectively. **Figure 2** shows that the risk of developing carotid artery plaques and fatty liver increases with higher tertiles of CHG, TYG, and AIP during the follow-up period.

**Figure 3:**
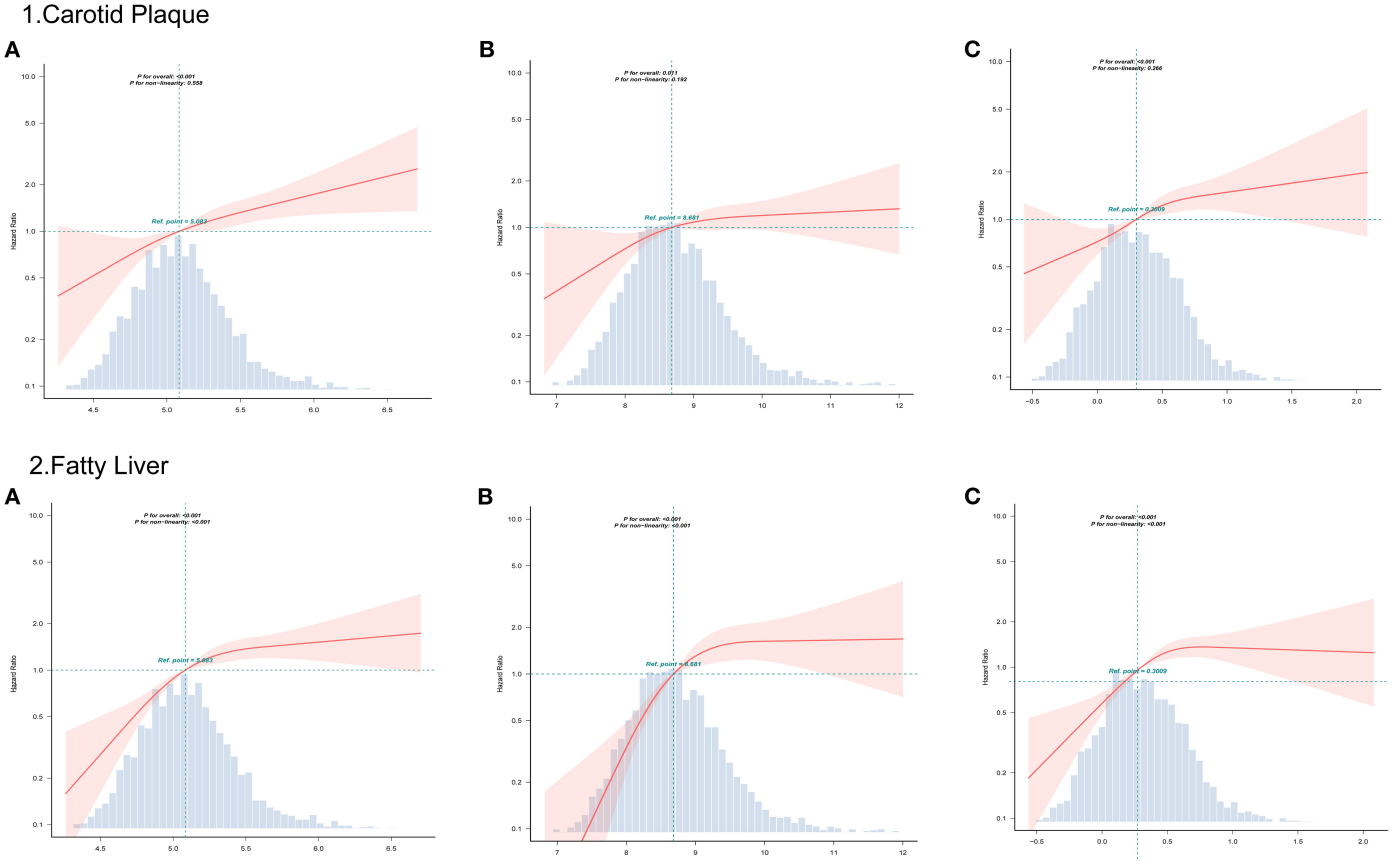
RCS curves analyzed the relationship between CHG **(A)**, TYG **(B)**, AIP **(C)**, and outcomes risk.

The fitted curves in **Figure 3** indicate a linear relationship between CHG, TYG, AIP, and carotid artery outcome risk (*P* for non-linear < 0.001). The three indices exhibited a linear relationship with fatty liver outcome, showing an inverted L-shaped pattern. In the non-linear relationship, no inflection points were found that could be further used for stratified analysis.

### ROC curves between CHG, TYG, and AIP and outcome risk events

**Figure 4:**
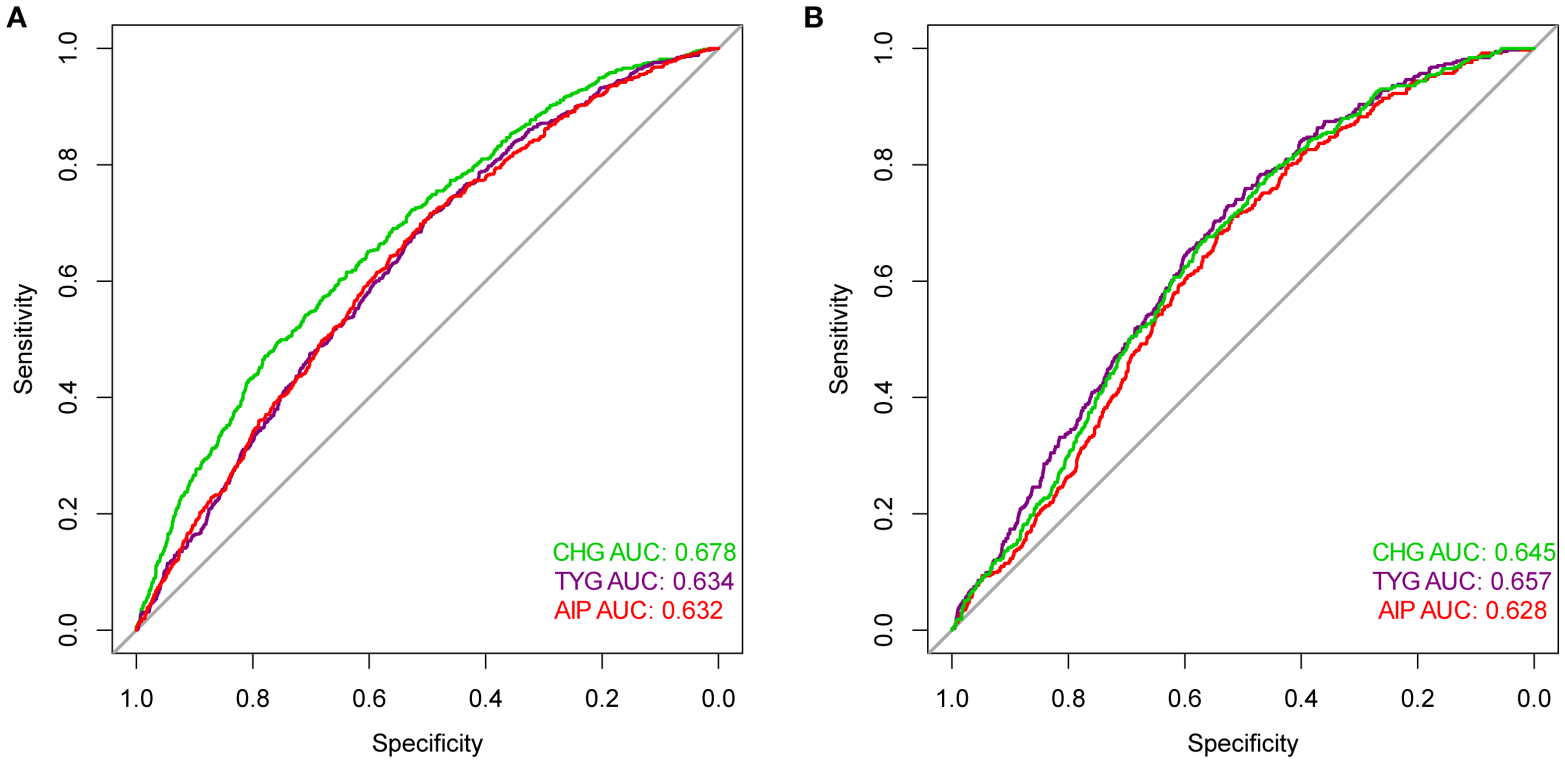
ROC curves compared the predictive efficacy of the CHG, TYG, AIP for outcomes [**(A)** Carotid plaque; **(B)** Fatty liver] risk events.

**Figure 4** displays the predictive performance among the three indicators for carotid plaque and fatty liver risk. **Supplementary Table S2** confirms that the AUC differences among them passed the significance test. In terms of carotid plaque prediction, the AUC for the CHG index was 0.678, while TYG and AIP were 0.634 and 0.632, respectively. For fatty liver outcomes, TYG showed the highest AUC (0.657), followed by CHG (0.645), both outperforming AIP (0.628).

### Subgroup analysis

**Figure 5:**
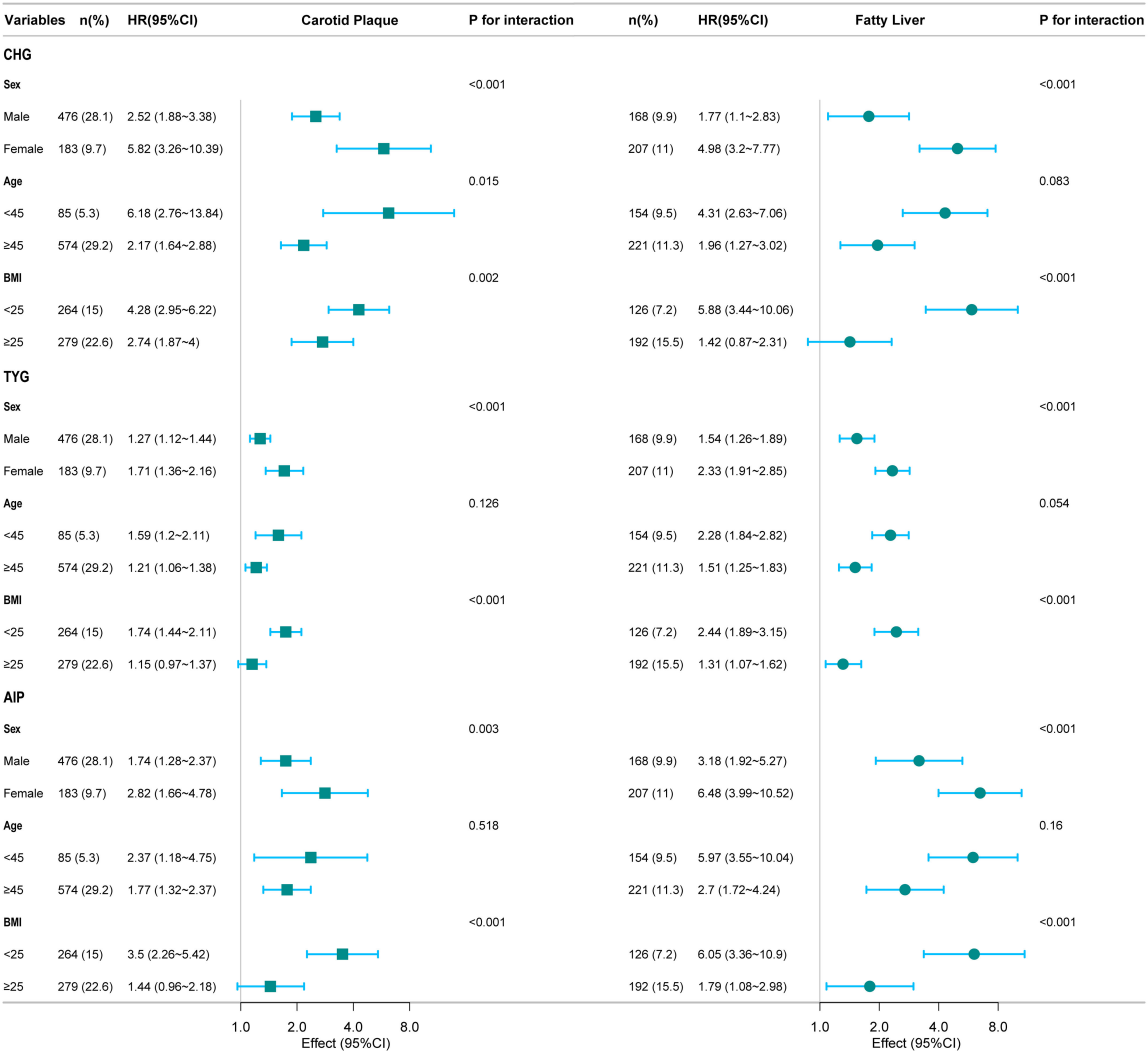
Forest plot of CHG, TYG, AIP in predicting outcomes risk.

Subgroup analysis revealed that gender and BMI interacted with the relationship between the three indices with carotid plaque and fatty liver outcomes (**Figure 5**). In addition, age interacted with the relationship between CHG and carotid plaque outcomes.

## Discussion

The study revealed the following findings. First, CHG, TYG, and AIP were all positively correlated with increased risks of carotid plaque and fatty liver. Second, the three indices showed a linear relationship with carotid plaque outcomes and a nonlinear (inverted L-shaped) relationship with fatty liver outcomes. Third, CHG demonstrated superior predictive ability for carotid plaque outcomes, whereas TYG demonstrated better performance for fatty liver outcomes. Fourth, subgroup analysis revealed that the associations between the indices and outcomes were modified by gender and BMI.

Our subgroup analysis found that these metabolic indicators were more significantly associated with carotid plaque and fatty liver in women and people with higher BMI. Past research has shown that factors such as obesity, lifestyle, and environment can all influence the occurrence and development of carotid artery plaques and fatty liver disease (23, 24). BMI may be more likely to affect the subclinical early stages of carotid plaque, with each standard deviation increase resulting in an 11% increase in plaque burden (25). YU et al. conducted a cross-sectional study specifically targeting steelworkers in northern China to investigate the relationship between obesity metabolism and carotid artery health. Research has found that, among obese patients, participants with unhealthy metabolic phenotypes have a significantly higher risk of developing carotid plaques than those with healthy metabolic phenotypes (26). It is clear that BMI alone cannot fully capture metabolic status, and lifestyle and genetics also play a key role in the progression of fatty liver disease (27). Sex, as the key genetic factor, is decisive for metabolic traits. Compared to men, the reduced circulating levels of sex hormones in women at the onset of menopause cause them to be more susceptible to impaired insulin sensitivity and impaired lipid regulation, among other things, which increase the risk of CVD (28). Gong found that the correlation between multiple IR indicators and metabolic disease was more pronounced in women (29).

The CHG was first proposed by Mansoori et al. as an index to improve the simplicity of diagnosing type 2 diabetes. Compared with TYG, the CHG index has higher specificity (16). Subsequent studies demonstrated its advantages in predicting the risk of diabetic nephropathy and CVD (15, 30). Research on CHG remains limited for metabolic-related and cardiovascular-related diseases. In contrast, substantial evidence links elevated TYG levels to increased risks of CVD and cerebrovascular events (heart failure, coronary heart disease, stroke) (31–33). A three-year longitudinal study identified that TYG can act as both a predictor and dose-response indicator for carotid plaque (34). TYG also correlates with prognosis in metabolic diseases (including diabetes, insulin resistance and fatty liver) (14, 35). A cohort study by NAGALA examined the correlation and predictive ability of 15 obesity and lipid-related indicators with fatty liver disease and found that the TyG index had the strongest correlation and the best predictive performance (36). Our study also showed that among the three indices, TYG had better predictive performance for fatty liver. Additionally, Mo et al. reported higher hazard ratios for CHG than TYG for CVD risk, which aligns with our carotid plaque results (15).

Compared with the lipid-only AIP index, CHG and TYG indices (incorporating glucose) demonstrated stronger correlations and predictive ability for carotid plaque and fatty liver outcomes. The shared metabolic disorder underlying these conditions involves an IR-triggered pathological network (37). IR not only causes peripheral glucose uptake disorders and increased hepatic glucose output, but also triggers the influx of free fatty acids (FFA) into the liver through abnormal lipolysis in adipose tissue, forming a lipotoxic microenvironment (38). Under metabolic stress, hepatic activation occurs through dual pathways: 1) hepatocyte FFA accumulation triggers oxidative/ER stress, stimulating Kupffer cells to release pro-inflammatory cytokines (IL-6, TNF-α) that amplify systemic inflammation via portal circulation; 2) steatotic hepatocytes secrete aberrant adipokines (reduced adiponectin, elevated resistin) synergizing with visceral fat-derived adipokines to promote atherosclerosis (39, 40). Hepatocyte-derived resistin activates NF-κB to drive monocyte vascular infiltration, forming a “metabolic-inflammatory-vascular injury” cycle with counterregulatory GLP-1 elevation. This liver-vascular axis explains the superior predictive value of integrated markers like CHG/TYG: they concurrently capture IR’s triad of hepatic glucose dysregulation, adipose lipolysis, and endothelial dysfunction, thus better reflecting the shared pathogenesis of fatty liver and atherosclerosis than lipid-only indices.

A particularly noteworthy observation in our analysis was the distinct dose-response relationship patterns between the metabolic indices and the two clinical outcomes. While all three indices exhibited a linear association with carotid plaque risk, their relationships with fatty liver development demonstrated a characteristic inverted L-shaped, nonlinear pattern upon RCS analysis. Each incremental increase in CHG, TYG, or AIP contributes additively to atherosclerotic risk, consistent with the known progressive nature of vascular endothelial dysfunction, lipid infiltration, and inflammatory activation in atherosclerosis (41, 42). In contrast, the risk of fatty liver disease exhibits an inverted L-shaped correlation pattern, suggesting the potential presence of a threshold phenomenon driven by complex mechanisms. Once a critical threshold of hepatocyte steatosis is exceeded, additional lipid influx may be diverted to ectopic deposition or undergo alternative metabolic fates rather than proportionally increasing visible steatosis (43). Mitochondrial β-oxidation, VLDL deposition, and activation of adaptive hepatocyte signaling pathways (FGF21, adiponectin) can systemically regulate lipid metabolism and insulin sensitivity, potentially activating compensatory homeostasis mechanisms triggered by severe lipid overload (44–46). Another interpretation is that these composite indicators exhibit high sensitivity in detecting the initial stages of insulin resistance and dyslipidemia. However, once specific metabolic thresholds are crossed and other pathophysiological mechanisms dominate disease progression, their discriminatory power significantly diminishes.

The CHG and TYG indices are readily acquired from routine metabolic panels and show significant associations with target organ damage, supporting their potential utility in clinical assessment. Incorporating them into early risk stratification as supplementary indicators alongside ultrasound-based screening strategies may aid in optimizing healthcare resource allocation. Moreover, these indices could help inform therapeutic decision-making. Identifying high-risk patients using CHG/TYG may justify earlier intensification of therapy, including novel agents such as PCSK9-targeting RNA-based therapeutics for robust lipid management (47, 48). Future studies should explore whether reduction in these indices following intervention correlates with regression of subclinical disease, potentially positioning CHG/TYG as dynamic biomarkers for treatment monitoring.

This study has several advantages. First, it represents the first investigation into CHG’s relationship with both carotid plaque and fatty liver. Additionally, CHG, AIP, and TYG indices were compared, revealing novel insights on glucose-lipid versus lipid-only assessment for outcome prediction. However, limitations exist. First, the findings from this single-center cohort of steelworkers may not fully represent the general population and require further validation in population-based cohort studies. Second, despite adjusting for confounding factors, inevitable missing data (comorbidities, medication records and alcohol consumption) may still lead to potential bias. Third, the study’s limitation to a Chinese population restricts its generalizability across ethnic groups, necessitating further research in different countries and populations.

## Conclusion

Collectively, CHG, TYG, and AIP demonstrated positive associations with carotid plaque and fatty liver risks, with CHG showing superior predictive performance for carotid plaque outcomes and TYG exhibiting optimal prediction for fatty liver.

## Data Availability

The original contributions presented in the study are included in the article/**Supplementary Material**. Further inquiries can be directed to the corresponding author/s.
